# Extraction of Dihydroquercetin from *Larix gmelinii* with Ultrasound-Assisted and Microwave-Assisted Alternant Digestion

**DOI:** 10.3390/ijms13078789

**Published:** 2012-07-16

**Authors:** Chunhui Ma, Lei Yang, Wenjie Wang, Fengjian Yang, Chunjian Zhao, Yuangang Zu

**Affiliations:** State Engineering Laboratory for Bio-Resource Eco-Utilization, Northeast Forestry University, Harbin 150040, China; E-Mails: mchmchmchmch@163.com (C.M.); ylnefu@163.com (L.Y.); yangfj@nefu.edu.cn (F.Y.); zcjsj@163.com (C.Z.)

**Keywords:** *Larix gmelinii* wood, dihydroquercetin, ultrasound and microwave assisted alternant extraction, peroxide value, scanning electronic microscope

## Abstract

An ultrasound and microwave assisted alternant extraction method (UMAE) was applied for extracting dihydroquercetin (DHQ) from *Larix gmelinii* wood. This investigation was conducted using 60% ethanol as solvent, 1:12 solid to liquid ratio, and 3 h soaking time. The optimum treatment time was ultrasound 40 min, microwave 20 min, respectively, and the extraction was performed once. Under the optimized conditions, satisfactory extraction yield of the target analyte was obtained. Relative to ultrasound-assisted or microwave-assisted method, the proposed approach provides higher extraction yield. The effect of DHQ of different concentrations and synthetic antioxidants on oxidative stability in soy bean oil stored for 20 days at different temperatures (25 °C and 60 °C) was compared. DHQ was more effective in restraining soy bean oil oxidation, and a dose-response relationship was observed. The antioxidant activity of DHQ was a little stronger than that of BHA and BHT. Soy bean oil supplemented with 0.08 mg/g DHQ exhibited favorable antioxidant effects and is preferable for effectively avoiding oxidation. The *L. gmelinii* wood samples before and after extraction were characterized by scanning electron microscopy. The results showed that the UMAE method is a simple and efficient technique for sample preparation.

## 1. Introduction

*Larix gmelinii* is a deciduous tree primarily distributed in northeast China, north Sakhalin and east Siberia. It occupies nearly 55% of Great Khingan and Lesser Khingan, China [1]. With its physical properties, such as rigidness, straight grain and corrosion resistance, *L. gmelinii* has been widely used for furniture and buildings. As a result, large quantities of logging slashes (stump, branch and root), bucking residues and processing residues (sawdust, crushed veneer, wood core, shavings) are produced each year as a side product. Hence, there is a growing interest in the comprehensive utilization of *L. gmelinii* resource.

Dihydroquercetin (DHQ, taxifolin), 3,3′,4′,5,7-pentahydroxiflavanon, is a kind of flavanonol [2], is an active compound isolated from the xylem of *L. gmelinii* [3]. It is also found in açaí palm, *Rosa davurica* stalk*,* milk thistle seeds and red onion. DHQ has a positive effect on human health as it prevents accumulation of free radicals [4,5], has antiradiation [6], antiviral [7,8], antitumor [9–11] activities, influences the physical properties of lipids in the biological membranes [12], ameliorates cerebral ischemia-reperfusion injury [13] and activates the formation of collagen fibers [14]. Application of DHQ is quite wide in the production of different categories of products. In general, DHQ can be used as a natural antioxidant additive in the food industry [3].

The extraction of DHQ has been accomplished using several extraction methods in the past. These include heating, boiling or refluxing with water [3,15] and refluxing extraction with ethanol and acetone [16,17]. Unfortunately, these methods involve long and tedious procedures employing large quantities of expensive toxic organic solvents. In recent years, the development and use of environmentally friendly methods has become increasingly popular. Much wider attention has been given to applications of ultrasound [18] and microwave [19] for DHQ extraction. Compared with conventional solvent extraction, the use of ultrasound makes the extraction of valuable compounds more efficient by means of shorter time frames and lower extraction temperatures [20]. The possible benefits of ultrasound in extraction are mass transfer intensification, cell disruption, improved penetration and capillary effects [21]. Ultrasound is currently employed to extract such pharmacologically active compounds as lignans [22], coumarins [23], and alkaloids [24,25] from plant materials. As many researchers have already stated in numerous published papers, microwave irradiation produces efficient internal heating for most chemical reactions, delivering energy exactly where it is needed, even under exothermic conditions [26]. This energy is mainly used for organic synthesis and sample digestion, extraction, and dissolution applications. Microwave has shown potential in the extraction of various useful substances from plant samples, such as phenolic compounds [27], proanthocyanidins [28], lignans [29], and alkaloids [30]. MAE techniques share the advantages of is being green, increasing yields and reducing reaction times, thus saving energy [31]. Consequently, combining ultrasonic with microwave is a complementary technique and may have several advantages. However, there are few reports of the application of ultrasound and microwave assisted alternant extraction (UMAE) of DHQ from plant materials.

The primary aim of the present study was to develop an effective, rapid ultrasound and microwave assisted alternant approach for the extraction of DHQ from *L. gmelinii* wood, and to compare the results with conventional extraction methods. It was found that parameters including the ethanol concentration, soaking time, solid–liquid ratio, extraction cycle, ultrasound and microwave power and time were influential on the extraction yield, and these parameters were optimized systematically. The effect of the extracts and DHQ of different concentrations on oxidative stability in soy bean oil were evaluated. Furthermore, the ultra-structural changes of plant materials were analyzed by scanning electron microscopy (SEM).

## 2. Results and Discussion

### 2.1. Common Factors of UAE and MAE

#### 2.1.1. Extraction Solvent

**Figure 1:**
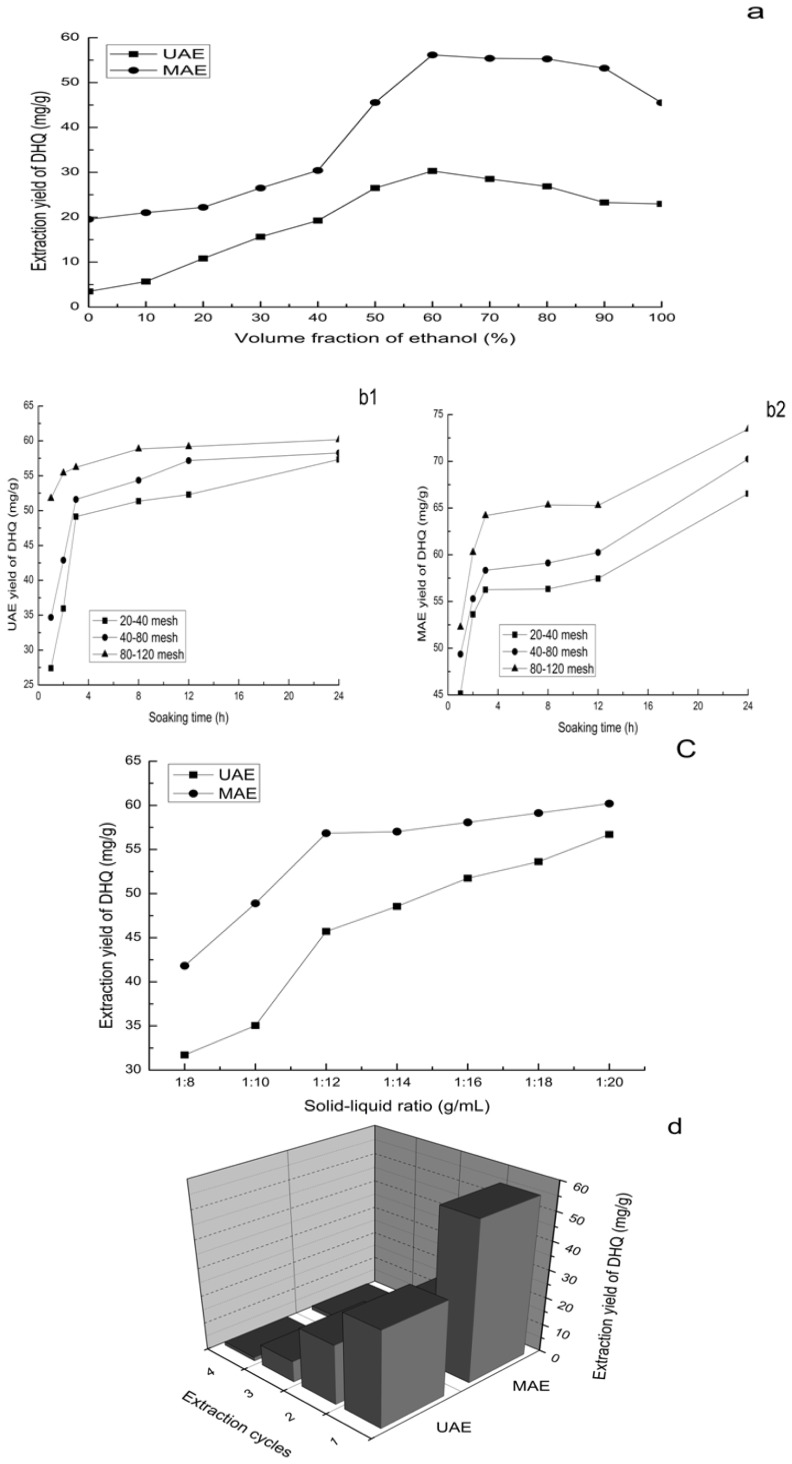
Effect of common factors on extraction yield of dihydroquercetin (DHQ). (**a**) Effect of volume fraction of ethanol on extraction yield; (**b**) Effect of soaking time on extraction yield; (**c**) Effect of solid-liquid ratio on extraction yield; (**d**) Effect of extraction cycles on extraction yield.

As we all know, ethanol with different concentration is the most commonly extracted solvent, because of its strong ability to penetrate the plant cell wall, lower boiling point (related to industrial energy consumption and solvent recovery), low cost and non-toxicity. The ethanol concentration is an important factor, because the solubility biological small molecules (DHQ) in ethanol solution determine the extraction yield of DHQ. *L. gmelinii* wood flour 30.0 g was extracted by ultrasound or microwave for 30 min with different ethanol concentration (10%–100%), the solid-liquid ratio was 1:10. The experiments were repeated three times and averaged. As can be seen from Figure 1a, with the ethanol concentration increased, the yield of DHQ was showed an upward trend, and the yield of DHQ was up to the maximum value when the ethanol concentration was 60%. While the ethanol concentration was greater than 60%, the yield of DHQ decreased slightly. So, 60% ethanol is selected for the extraction of DHQ.

#### 2.1.2. Soaking Time

The role of infiltration in the extraction process is to make the raw materials fully run-up under the condition of non-energy consumption, which can help the dissolution of small molecules in a solution. The treatment time of infiltration is longer and the run up is better. However, long infiltration may cause moldy materials (if the solvent is water), long extraction cycles and low extraction efficiency. In this experiment, 30.0 g wood flour of different particle size was soaked with 60% ethanol for 0, 1, 2, 3, 8, 12 and 24 h, respectively. The solid-liquid ratio was 1:10, and the effect of soaking time and the particle size of raw materials on the yield of DHQ was studied. In Figure 1b, the DHQ extraction yields of UAE and MAE increased with the soaking time for the first 3 h, and were almost unchanged after 3 h. However, in Figure 1(b2), the DHQ extraction yield of MAE increased dramatically with soaking time as it increased from 12 h to 24 h. This is because a long infiltration time is conducive to full dissolution of DHQ molecule under the condition of being heated. However, after a long infiltration time, the wood flour is difficult to filter. In order to save time and maximize infiltration, the soaking time is 3 h for further experiments.

#### 2.1.3. Solid–Liquid Ratio

Large solvent content may cause complex procedures and unnecessary waste, increasing the energy consumption of recycling, while small solvent content may cause incomplete extraction. To evaluate the effect of the solid-liquid ratio, a series of extractions were carried out with different solid-liquid ratios (1:8, 1:10, 1:12, 1:14, 1:16, 1:18 and 1:20 g/mL). Figure 1c indicated that the extraction yield of DHQ increased following the increase of the solvent volume before the solid-liquid ratio reached 1:12, and was then not significantly influenced by further increase of the solvent amount. Therefore, a solid-liquid ratio of 1:12 was determined and then used in the further studies.

#### 2.1.4. Extraction Cycles

Extraction cycles are a crucial factor in increasing the extraction yield of DHQ, but too many times, will lead to excessive dissolution of impurities, resulting in undue burden to the subsequent separation and purification process. From Figure 1d, the total four-times extraction yield of UAE and MAE were 64.06 and 80.66 mg/g, respectively, and the total extraction yield of four times was defined as 100%. Each of the extraction yields of DHQ were then compared with the total extraction yield. Each-time extraction yield of UAE were 33.95, 21.23, 7.60, and 1.27 mg/g, respectively. The extraction yield sum of 2 times had reached more than 85%, and the extraction yield sum of 3 times was about 98%. Each-time extraction yield of MAE were 56.16, 18.65, 4.32, and 1.53 mg/g, respectively. The extraction yield sum of 2 times had reached to 92.74%, and the extraction yield sum of 3 times was more than 98%. Considering the relevant factors, including time, solvent and energy consumption, extraction 3 times was more reasonable.

### 2.2. Core Factors of UMAE

#### 2.2.1. Extraction Dynamics of DHQ with UAE and MAE

In order to investigate the effect of extraction time on the extraction yield of DHQ with UAE and MAE, respectively, the process was performed in ultrasound unit and microwave oven under the optimized conditions. Thirty grams of dried sample was mixed with 60% ethanol soaked for 3.0 h, and then extracted for 60 min (UAE) and 30 min (MAE), respectively. The power of UAE and MAE were 250 W and 700 W, respectively. The solid-liquid ratio was 1:12, and the yields of DHQ were tested every 10 min and 5 min, respectively.

**Figure 2:**
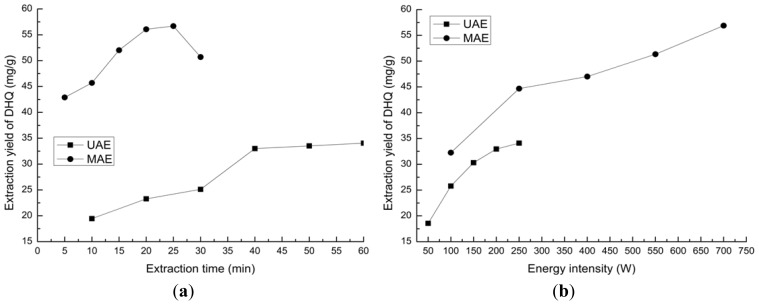
Effect of core factors on extraction yield of DHQ. (**a**) Effect of extraction time on extraction yield; (**b**) Effect of energy intensity on extraction yield.

From Figure 2a, it can be seen that the yield of DHQ with MAE was much higher than that with UAE. As the extraction time increased, the yield of DHQ increased at first. Then, after UAE 40 min, the extraction yield of DHQ was almost unchanged, and after MAE 20 min, the yield had even declined. This may result in DHQ degradation with long microwave irradiation times. Therefore, 40 min ultrasound treatment and 20 min microwave irradiation time were selected as the optimization conditions for extraction DHQ.

#### 2.2.2. Energy Intensity of UAE and MAE

To examine the effect of the ultrasound power on the extraction yield of DHQ, extractions were carried out with a constant time for ultrasound treatment of 40 min at 50, 100, 150, 200, and 250 W, respectively. Figure 2b indicated that the average extraction yield was significantly influenced when the ultrasound power was increased. Therefore, the 250 W ultrasound powers (the maximum value of ultrasound unit) satisfied the conditions for extracting of DHQ.

In order to study the effect of the microwave power on the extraction yield, extractions were carried out with 20 min for microwave treatment at 100, 250, 400, 550 and 700 W, respectively. Figure 2b indicated that the average extraction yield was increased with the irradiation power increasing. Thus, the 700 W (the maximum value of microwave oven) microwave power satisfied the conditions for DHQ extraction.

### 2.3. Alternant Digestion by UAE and MAE

**Figure 3:**
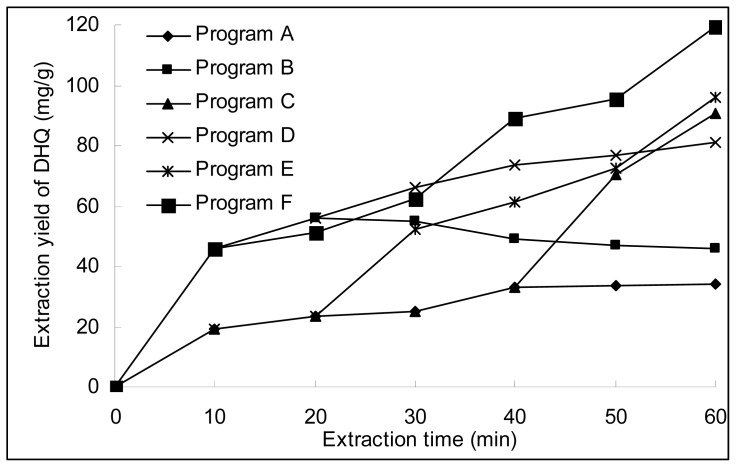
Alternant extraction by UAE and MAE.

The alternant test program and the extraction yield of DHQ with each program are shown in Table 1A–F. From Figure 3, the yield of DHQ extracted by UMAE (C–F) is higher than that single extraction by UAE (A) or MAE (B), although the extraction time is the same. The extraction yield of program F is 119.6 ± 2.9 mg/g, highest of all, and it also higher than the maceration and reflux extraction. The results indicated that alternant extraction with UMAE could obtain the higher yield of DHQ than that extracted by UAE or MAE separately.

### 2.4. Comparison with Different Extraction Methods

#### 2.4.1. Comparison the Extraction Yield of DHQ with Different Extraction Methods

As can be seen from Table 1, the extraction yield of DHQ with item F was 119.6 ± 2.9 mg/g, better than other comparison methods. Conversely, reflux extraction is a time-saving method, but it has energy consumption in the process of heating. The extraction yield of DHQ with 60% ethanol reflux extraction 4 h was 90.0 ± 2.8 mg/g. Apparently, water is the most common and inexpensive solvent, therefore pure water is always selected as the co-solvent in various extraction processes. The extraction yield of DHQ with water stirring extraction for 8 h at 50 °C was only 35.6 ± 1.8 mg/g, which was similar to the extraction yield of DHQ with water reflux extraction for 4 h (35.0 ± 1.4 mg/g). Compared to above extraction methods, alternant digestion by UAE and MAE was a better extraction method of DHQ.

#### 2.4.2. Comparison Environmental Impact with Different Extraction Methods

The most common method for extraction of DHQ from *L. gmelinii* is reflux extraction using 60% ethanol. The energy required to perform the reflux extraction method is 1.00 kW. Table 1 shows that, not only is the extraction yield of DHQ by UMAE (119.6 ± 2.9 mg/kg) higher than that by reflux extraction (90.0 ± 2.8 mg/kg), but also the total time of energy consumption dramatically reduces (from 4 h to 1 h) and the carbon dioxide rejected in the atmosphere reduces (from 3200 g CO_2_ to 320 g CO_2_ rejected). The reduced cost of DHQ extraction is clearly advantageous for the UMAE method in terms of time and energy. The energy consumption time required by reflux extraction is 4 h. The energy powers required are 1.00 kW/h for reflux extraction, 0.7 kW/h for MAE, and 0.25 kW/h for UAE, respectively. Regarding environmental impact, the calculated quantity of carbon dioxide rejected in the atmosphere of reflux extraction (3200 g CO_2_) is much higher than that of UMAE (320 g CO_2_). These calculations have been made according to the literature [32]: to obtain 1 kW·h from coal or fuel, 800 g of CO_2_ will be rejected in the atmosphere during combustion of fossil fuel. Therefore, UMAE can be suggested as an environmentally friendly extraction method, which avoids the use of organic solvents. UMAE can also be offered for the production of larger quantities of DHQ by applying the existing large-scale ultrasound-microwave extractors instead of the conventional reflux extractors, with the improvement and development of ultrasonic and microwave devices.

### 2.5. Morphology by SEM

**Figure 4:**
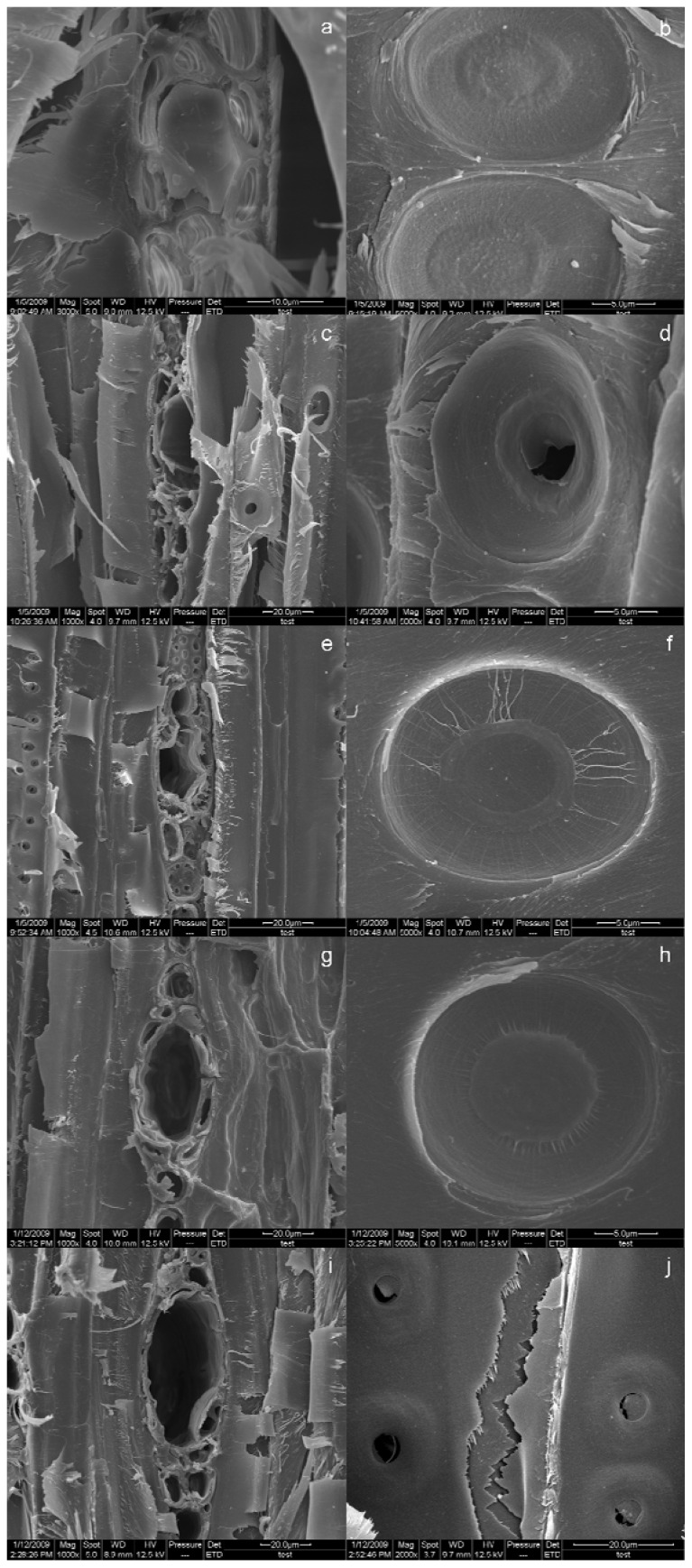
Morphology analysis. Scanning electron microscopy (SEM) images of resin ducts and pits of (**a**, **b**) raw materials, (**c**, **d**) the materials treated by program A, (**e**, **f**) the materials treated by program B, (**g**, **h**) the materials treated by program E, and (**i**, **j**) the materials treated by program F.

The morphological changes of the materials structure extracted by different methods were observed in SEM. DHQ is a flavanonol, and it is difficult to be observed directly in the wood structure. In the structure of *L. gmelinii* wood material, the resin ducts are clearly visible, and through observing the resin amount in resin ducts, to compared the effect of different extraction methods. Because resin is a kind of waxy substance, and difficult to extract, simply observing the amount of resin after extraction can help us determine which method is better complete [33,34]. As can be seen from Figure 4a,b, the resin ducts were filled with resin in the raw material structure, and the surface of pits was smooth. It can also be seen that the resin in resin ducts had been extracted basically completely by UAE (Figure 4c,d) or MAE (Figure 4e,f), and the pit membrane had a smooth surface without rupture with MAE, but the pit membrane had been broken with UAE, indicating that UAE has oscillations of greater mechanical strength. As can be seen from Figure 4g–j, not only had the resin been extracted basically completely and the resin ducts shriveled by UMAE, but the pit membrane had also been ruptured and many cracks occurred between the pits. Therefore, the unique physical characteristics of ultrasound can induce broken or deformed plant cell tissue, so that the active ingredient was extracted more rapidly and with lower power consumption; and the thermal effects with MAE can ensure that the active ingredients are dissolved completely in a short period of time, saving time and energy.

### 2.6. Antioxidant Activity of DHQ

#### 2.6.1. Peroxide Value (POV) after Addition of DHQ

POV is an important parameter of fats and oils, and DHQ as a kind of food additive that shows a good antioxidant effect. The changes in POV during storage of soy bean oil at 25 °C and 60 °C for 20 days after addition of DHQ were investigated and given in Tables 2,3. Lower POV shows a better resistance to oxidation; by increasing the dosage of DHQ, the antioxidant activity also increased. However, excessive dosage will result in unnecessary waste. It is evident from these results that, as the concentration of DHQ increased, inhibitory effects on POV also increased considerably, but when the concentration of DHQ was higher than 0.08‰, the inhibitory effects on POV was not increased significantly, and the optimal addition dosage of DHQ is 0.08‰ (the mass ratio of DHQ and soy bean oil). The addition of 0.08‰ DHQ caused significant reduction in POV from 0.090 ± 0.002 (control) to 0.146 ± 0.003 and 0.559 ± 0.007 meq/kg, during 20 days, storage at 25 °C and 60 °C, respectively.

#### 2.6.2. Comparison of Antioxidant Activity of Synthetic Antioxidants and DHQ

The changes in POV during storage of soy bean oil at 25 °C and 60 °C after addition of synthetic antioxidants are given in Tables 4 and 5. The addition dosage of synthetic antioxidants (BHA and BHT) is 0.08‰ (the mass ratio of antioxidants and soy bean oil), the same as the addition dosage of DHQ. It is apparent from these results that addition of DHQ, BHA and BHT retarded the development of rancidity in soy bean oil, but DHQ resulted in a slightly better protection than BHA and BHT. The POV of BHA and BHT decreased from 0.084 ± 0.002 and 0.091 ± 0.002 (control) meq/kg to 0.177 ± 0.003, 0.203 ± 0.003 meq/kg after 20 days’ storage at 25 °C, and to 1.032 ± 0.009, 1.380 ± 0.005 meq/kg after 20 days’ storage at 60 °C, respectively. Briefly, as antioxidant storage at 25 °C and 60 °C, the antioxidant activity order is DHQ > BHA > BHT.

## 3. Experimental Section

### 3.1. Materials

*L. gmelinii* wood was provided by Maoershan experimental forest farm of Northeast Forestry University (Heilongjiang, China), and identified by Shaoquan Nie from State Engineering Laboratory for Bio-Resource Eco-Utilization, Northeast Forestry University. The samples sieved by 20–120 mesh after crushing by FZ 102 plant grinder (Taisite, Tianjin, China). DHQ standard (98% purity) was purchased from the National Institute for the Control of Pharmaceutical and Biological Products (Beijing, China). Butylated hydroxyanisole (BHA, ≥99%) and butylated hydroxytoluene (BHT, ≥99%) were purchased from Sigma-Aldrich (St. Louis, MO, USA), Acetonitrile and acetic acid of HPLC grade were purchased from J&K Chemical Ltd. (Beijing, China). Refined, bleached, and deodorized soy bean oil was obtained from Jiusan Oils & Grains Industries Group Co., ltd (Heilongjiang, China) and was kept in a refrigerator at low temperature (4 °C). It contained Vitamin E 52 mg/kg and no synthetic antioxidants. The rest of the solvents and chemicals used in this study were of analytical grade and purchased from Beijing Chemical Reagents Co. (Beijing, China). Deionized water was purified by a Milli-Q water purification system (Millipore, MA, USA). All solutions prepared for HPLC were filtered through 0.45 μm membranes (Guang Fu Chemical Reagents Co. Tianjin, China) before use.

### 3.2. Methods

#### 3.2.1. HPLC Analysis Conditions

The HPLC system consists of a Waters 717 automatic sample handling system composed of series HPLC system equipped with 1525 Bin pump, 717 automatic column temperature control box and 2487 UV-detector (Waters, USA). Chromatographic separation was performed on a HiQ sil-C18 reversed-phase column (4.6 mm × 250 mm, 5 μm, Kya Tech) for the determination of DHQ. For HPLC analysis, acetonitrile-water-acetic acid (18:82:0.1, *v*/*v*/*v*) was used as the mobile phase with 1.0 mL/min flow rate, 10 μL injection volume, and 25 °C column temperature. The absorbance was measured at a wavelength of 294 nm for the detection of DHQ, the run time was 30 min, and the retention time of DHQ was 24 min. Corresponding calibration curves for DHQ is *Y* = 3.1755 × 10^7^*X* + 2.5993 × 10^4^ (*r* = 0.9999). A good linearity was found for DHQ in the range of 0.0312–0.5000 mg/mL.

#### 3.2.2. Alternant Digestion by UAE and MAE

First, the common factors of UAE and MAE by single-factor experiment with ultrasonic bath (KQ-250DB, Kunshan Ultrasonic Co. Ltd., China) and microwave oven (PJ21C-AU, Glanz, China) were optimized, and then the core factors of UAE and MAE were optimized, respectively. The common factors are included the volume fraction of ethanol, soaking time, solid-liquid ratio, and extraction times. The core factors are included extraction time and energy intensity of UAE and MAE, respectively. The alternant test program is shown in Table 1A–F.

#### 3.2.3. Comparison Extraction Methods

The conventional extraction methods of DHQ are including maceration extraction with 60% ethanol at room temperature for 24 h, reflux extraction with 60% ethanol for 2 h, stirring extraction with water for 8 h at 50 °C and reflux extraction with water for 4 h (in Table 1G–J). The yields of DHQ with the various extraction methods were compared.

#### 3.2.4. Scanning Electronic Microscopy

Microstructure analysis was performed using SEM (FEI QUANTA200, The Netherlands). The sample surfaces were sputter coated with a thin layer of gold using an SCD 005 sputter coater (BAL-TEC, Switzerland), to provide electrical conductivity.

#### 3.2.5. Assessment of Antioxidant Activity of DHQ

DHQ powder which was extracted by UMAE according to program F, as an antioxidant, was tested by the determination of POV during storage of soy bean oil at 25 °C and 60 °C. The POV (meq/kg of oil) was measured by titration with sodium thiosulphate, using starch as indicator, as described in Wang *et al.* [35,36]. Three grams of oil samples was dissolved in a blended solution of 30 mL chloroform-glacial acetic acid (2:3, *v*/*v*). A saturated solution of potassium iodide (1 mL) was added, airtight and shaken by hand for 0.5 min, and then kept in the dark for another 3.0 min. After the addition of 100 mL deionized water, the mixture was shaken, and immediately titrated against sodium thiosulphate (0.002 M) until the yellow color almost disappeared. Then, about 1.0 mL of starch indicator (0.1%) solution was added. Titration was sustained until the blue color just disappeared as the end point. A blank was also determined under similar conditions. POV was calculated as follows:

(1) $$\text{POV }(\text{meq}/\text{kg})=(V_{1}-V_{2})\times c\times 0.1269\times 78.8/m$$

where *V*_1_ is the reagent blank consumption volume of sodium thiosulfate standard solution, mL; *V*_2_ is the sample consumption volume of sodium thiosulfate standard solution, mL; *c* is the concentration of sodium thiosulfate standard solution, M; 0.1269 is the considerable iodine mass titrated with 1.00 mL sodium thiosulfate standard solution [*c* (Na_2_S_2_O_3_) = 1.00 M], g; 78.8 is conversion factor; *m* is the mass of oil sample, g. All determinations were carried out in triplicate and the statistical significance was evaluated using Excel and set at *p* < 0.05.

## 4. Conclusions

In summary, UMAE was applied for extracting DHQ from *L. gmelinii* wood, using the extraction yield as a response value. This investigation was conducted using 60% ethanol as solvent, 1:12 solid to liquid ratio, and 3 h soaking time. The optimum treatment time was ultrasound-assisted 40 min, microwave-assisted 20 min, respectively, and the extraction was performed once. The extraction yields of DHQ were 33.0 ± 1.2 and 56.1 ± 1.5 mg/g respectively, while that of UMAE was 119.6 ± 2.9 mg/g, which is much higher than ultrasound- or microwave- extraction alone. By SEM observation, the ultrasound-assisted and microwave-assisted alternant effect of substance dissolution from wood cells was more obvious than conventional extraction methods. The antioxidant activity of DHQ was tested through the POV of soy bean oil. During the 20 days storage of soy bean oil after the addition of DHQ, POV of oil decreased from 0.090 ± 0.002 (control) meq/kg to 0.146 ± 0.003 and 0.559 ± 0.007 meq/kg, storage at 25 °C and 60 °C, respectively, and the optimal dosage of DHQ is 0.08‰ of the mass of soy bean oil. Compared with synthetic antioxidants, the antioxidant activity order is DHQ > BHA > BHT. The present results demonstrate that the UMAE is a simple, fast, and effective extraction method. Moreover, the UMAE method that was proposed in this work will be a promising environmentally friendly technique in sample preparation.

## Figures and Tables

**Table 1:** Experimental program of ultrasound assisted alternant extraction method (UAE), microwave assisted alternant extraction method (MAE) and comparison extraction methods.

Item	Alternant experimental program	Extraction yield (mg/g)	Environmental impact (g CO_2_ rejected)
A	UAE 60 min with 60% ethanol	34.0 ± 1.1	200
B	MAE 60 min with 60% ethanol	46.1 ± 1.2	560
C	UAE 40 min + MAE 20 min with 60% ethanol	90.6 ± 2.0	320
D	MAE 20 min + UAE 40 min with 60% ethanol	81.3 ± 3.3	320
E	UAE 20 min + MAE 10 min + UAE 20 min +	96.2 ± 3.1	320
	MAE 10 min with 60% ethanol		
F	MAE 10 min + UAE 20 min + MAE 10 min +	119.6 ± 2.9	320
	UAE 20 min with 60% ethanol		
G	Maceration extraction 24 h with 60% ethanol solution at room temperature	12.3 ± 3.0	/*
H	Reflux extraction 4 h with 60% ethanol solution	90.0 ± 2.8	3200
I	Water stirring extraction 8 h at 50 °C	35.6 ± 1.8	6400
J	Water reflux extraction 4 h	35.0 ± 1.4	3200

* For low extraction yield the quantity of carbon dioxide rejected in the atmosphere was not calculated.

**Table 2:** Peroxide Value (POV) of soy bean oil after addition of DHQ during storage at 25 °C.

Storage time (days)	POV of soy bean oil (added 0.00‰^a^ DHQ)	POV of soy bean oil (added 0.02‰ a DHQ)	POV of soy bean oil (added 0.04‰ a DHQ)	POV of soy bean oil (added 0.06‰ a DHQ)	POV of soy bean oil (added 0.08‰ a DHQ)	POV of soy bean oil (added 0.10‰ a DHQ)
0	0.092 ± 0.002	0.092 ± 0.002	0.091 ± 0.003	0.090 ± 0.002	0.090 ± 0.002	0.090 ± 0.002
5	0.113 ± 0.002	0.112 ± 0.003	0.102 ± 0.003	0.098 ± 0.002	0.093 ± 0.002	0.089 ± 0.002
10	0.116 ± 0.003	0.115 ± 0.003	0.109 ± 0.003	0.105 ± 0.002	0.103 ± 0.003	0.103 ± 0.002
15	0.163 ± 0.002	0.148 ± 0.003	0.136 ± 0.002	0.125 ± 0.003	0.110 ± 0.003	0.108 ± 0.002
20	0.218 ± 0.003	0.193 ± 0.003	0.172 ± 0.002	0.159 ± 0.003	0.146 ± 0.003	0.139 ± 0.002

a 0.00‰–0.10‰ is the ratio of the mass of DHQ and the mass of soy bean oil.

**Table 3:** POV of soy bean oil after addition of DHQ during storage at 60 °C.

Storage time (days)	POV of soy bean oil (added 0.00‰^a^ DHQ)	POV of soy bean oil (added 0.02‰ a DHQ)	POV of soy bean oil (added 0.04‰ a DHQ)	POV of soy bean oil (added 0.06‰ a DHQ)	POV of soy bean oil (added 0.08‰ a DHQ)	POV of soy bean oil (added 0.10‰ a DHQ)
0	0.092 ± 0.002	0.092 ± 0.002	0.091 ± 0.003	0.090 ± 0.002	0.090 ± 0.002	0.090 ± 0.002
5	0.124 ± 0.003	0.119 ± 0.003	0.109 ± 0.002	0.102 ± 0.003	0.096 ± 0.002	0.093 ± 0.002
10	0.231 ± 0.002	0.174 ± 0.003	0.159 ± 0.003	0.152 ± 0.003	0.136 ± 0.003	0.133 ± 0.002
15	1.265 ± 0.006	0.713 ± 0.005	0.516 ± 0.004	0.448 ± 0.005	0.355 ± 0.004	0.300 ± 0.002
20	2.143 ± 0.012	1.469 ± 0.010	1.112 ± 0.008	0.800 ± 0.009	0.559 ± 0.007	0.509 ± 0.005

a 0.00‰–0.10‰ is the ratio of the mass of DHQ and the mass of soy bean oil.

**Table 4:** POV of soy bean oil after addition of synthetic antioxidants during storage at 25 °C.

Storage time (days)	POV of soy bean oil (added 0.08‰ a DHQ)	POV of soy bean oil (added 0. 08‰ a BHA)	POV of soy bean oil (added 0. 08‰ a BHT)
0	0.090 ± 0.002	0.084 ± 0.002	0.091 ± 0.002
5	0.093 ± 0.002	0.096 ± 0.002	0.128 ± 0.002
10	0.103 ± 0.003	0.111 ± 0.002	0.142 ± 0.002
15	0.110 ± 0.003	0.138 ± 0.002	0.172 ± 0.002
20	0.146 ± 0.003	0.177 ± 0.003	0.203 ± 0.003

a 0.08‰ is the ratio of the mass of antioxidants and the mass of soy bean oil.

**Table 5:** POV of soy bean oil after addition of synthetic antioxidants during storage at 60 °C.

Storage time (days)	POV of soy bean oil (added 0. 08‰^a^ DHQ)	POV of soy bean oil (added 0. 08‰ a BHA)	POV of soy bean oil (added 0. 08‰ a BHT)
0	0.090 ± 0.002	0.084 ± 0.002	0.091 ± 0.002
5	0.096 ± 0.002	0.109 ± 0.002	0.131 ± 0.002
10	0.136 ± 0.003	0.177 ± 0.002	0.222 ± 0.002
15	0.355 ± 0.004	0.460 ± 0.003	0.688 ± 0.004
20	0.559 ± 0.007	1.032 ± 0.009	1.380 ± 0.005

a 0.08‰ is the ratio of the mass of antioxidants and the mass of soy bean oil.
